# Potential of selected fungal species to degrade wheat straw, the most abundant plant raw material in Europe

**DOI:** 10.1186/s12870-017-1196-y

**Published:** 2017-12-28

**Authors:** Jasmina Ćilerdžić, Milica Galić, Jelena Vukojević, Ilija Brčeski, Mirjana Stajić

**Affiliations:** University of Belgrade, Faculty of Biology, Takovska 43, Belgrade, 11000 Serbia

**Keywords:** Delignification, Laccases, Mn-oxidizing peroxidases, Mushrooms, Wheat straw

## Abstract

**Background:**

Structural component of plant biomass, lignocellulose, is the most abundant renewable resource in nature. Lignin is the most recalcitrant natural aromatic polymer and its degradation presents great challenge. Nowadays, the special attention is given to biological delignification, the process where white-rot fungi take the crucial place owing to strong ligninolytic enzyme system. However, fungal species, even strains, differ in potential to produce high active ligninolytic enzymes and consequently to delignify plant biomass. Therefore, the goals of the study were characterization of Mn-oxidizing peroxidases and laccases of numerous mushrooms as well as determination of their potential to delignify wheat straw, the plant raw material that, according to annual yield, takes the first place in Europe and the second one in the world.

**Results:**

During wheat straw fermentation, *Lentinus edodes* HAI 858 produced the most active Mn-dependent and Mn-independent peroxidases (1443.2 U L^−1^ and 1045.5 U L^−1^, respectively), while *Pleurotus eryngii* HAI 711 was the best laccase producer (7804.3 U L^−1^). Visualized bends on zymogram confirmed these activities and demonstrated that laccases were the dominant ligninolytic enzymes in the studied species. *Ganoderma lucidum* BEOFB 435 showed considerable ability to degrade lignin (58.5%) and especially hemicellulose (74.8%), while the cellulose remained almost intact (0.7%). Remarkable selectivity in lignocellulose degradation was also noted in *Pleurotus pulmonarius* HAI 573 where degraded amounts of lignin, hemicellulose and cellulose were in ratio of 50.4%:15.3%:3.8%.

**Conclusions:**

According to the presented results, it can be concluded that white-rot fungi, due to ligninolytic enzymes features and degradation potential, could be important participants in various biotechnological processes including biotransformation of lignocellulose residues/wastes in food, feed, paper and biofuels.

## Background

Lignocellulose, a structural component of plant biomass, is considered to be the most abundant renewable organic resource in terrestrial environments. It consists of a cellulose-hemicellulose matrix immersed in net of lignin, the most recalcitrant natural aromatic polymer [1]. Although various methodologies for the biomass delignification are available, they are commonly unsatisfactory due to high process cost, significant energy consumption and release of non-enviromentally friendly by-products [2]. Since chemical, physical, and physico-chemical biomass pre-treatments are characterized with all mentioned shortages, recently there is a great deal of interest for biological delignification [3]. The advantages of biological methods are significant delignification selectivity, low energy consumption, absence of toxic by-products and economical justification [4]. White-rot fungi take the crucial place in the biological biomass pre-treatment as they possess strong ligninolytic enzyme system containing lignin- and Mn-oxidizing peroxidases, laccases and some auxiliary enzymes [5]. The high efficiency of the enzymes is based on their strong oxidative activity and low substrate specificity and depends on fungal species/strain capacity, oxidative mechanism, lignocellulose nature and cultivation conditions [6]. The remarkable ligninolytic potential makes the white-rot mushrooms the main actors in various biotechnological processes, such as production of food, feed, paper, biofuel, textile, as well as soil and water remediation [7].

According to abundance, wheat straw takes the first place in Europe, with annual yield of 170 million tons, and the second one in the world [8, 9]. Therefore, it presents a promising, one of the cheapest and the most useful raw material for biotransformation in various products [6]. Since, bioconversion capacity varies depending on fungal species and/or strain, the objectives of this research were profiling of Mn-oxidizing peroxidases and laccases of selected mushrooms as well as determination of their wheat straw delignification potential.

## Methods

### Organisms and cultivation conditions

The cultures of studied species/strains were isolated from fruiting bodies collected in Serbia, Israel, Russia, Ukraine and England (Table 1), and maintained on Malt agar medium in culture collection of University of Belgrade - Faculty of Biology (BEOFB).

**Table 1:** The studied species/strains

Studied species	Strain code	Origin of strains
*Ganoderma lucidum*	BEOFB 435	Belgrade, Serbia
*Hypsizygus tessulatus*	BEOFB 910	from *Aesculus hippocastanum*, Serbia
*Lenzites betulinus*	BEOFB 500	from *Populus tremula*, Russia
*Lentinus edodes*	BEOFB 858	Poznan, Poland
*Pleurotus citrinopileatus*	HAI 435	Cultivated strain, England
*Pleurotus eryngii*	HAI 193	from *Stipa* sp., Kherson region, Ukraine
	HAI 711	from *Ferula* sp*.*, Tabor, Izrael
*Pleurotus ostreatus*	HAI 592	Russia
*Pleutorus pulmonarius*	HAI 573	Sochi, Russia
*Xylaria polymorpha*	BEOFB 1110	Botanical Garden, Belgrade, Serbia

The inoculation of 100 mL of synthetic medium (glucose, 10.0 g L^−1^; NH_4_NO_3_, 2.0 g L^−1^; K_2_HPO_4_, 1.0 g L^−1^; NaH_2_PO_4_ × H_2_O, 0.4 g L^−1^; MgSO_4_ × 7H_2_O, 0.5 g L^−1^; yeast extract, 2.0 g L^−1^; pH 6.5) with mycelial agar discs of 7-day old culture, incubation at room temperature (22 ± 2 °C) on rotary shaker (100 rpm), washing of obtained biomass by sterilized distilled water (three times) and its homogenization in laboratory blender were the main steps of inoculum preparation [10]. Solid-state cultivation was performed during 21 days, at 25 °C, in 100 mL flasks containing substrate composed of 2.0 g of wheat straw, as the carbon source, and 10.0 mL of the modified synthetic medium (without glucose) inoculated with 3.0 mL of the homogenized inoculum.

### Determination of enzyme activity and total protein content

According to the method of Stajić et al. [10], the produced extracellular enzymes were extracted by sample stirring with 50.0 mL dH_2_O on a magnetic stirrer at 4 °C for 10 min. The obtained extracts were centrifugated (4 °C, 3000 rpm, 15 min) and resulting supernatants were used for measurement of activities of Mn-oxidizing peroxidases [Mn-dependent peroxidase (MnP; EC 1.11.1.13) and Mn-independent peroxidase (MnIP; EC 1.11.1.16)] and laccases (EC 1.10.3.2), as well as the protein content, spectrophotometrically [CECIL CE2501 (BioQuest)]. Activities of Mn-oxidizing peroxidases and laccases were determined using phenol red (ε_610_ = 22,000 M^−1^ cm^−1^) and 2,2^′^-azino-bis-[3-ethylbenzothiazoline-6-sulfonate (ABTS) (ε_436_ = 29,300 M^−1^ cm^−1^), respectively, as the substrates. The amount of enzyme which transformed 1.0 μmol of substrate per minute was defined as the enzymatic activity of 1 U.

The content of total proteins (mg mL^−1^) was determined in the reaction mixture composed of Bradford reagent and sample, at λ = 595 nm, by standard curve obtained from known concentrations of bovine serum albumin [11].

All assays were carried out in quadruplicate and the results are expressed as the mean ± standard error.

### Electrophoresis

Mini IEF Cell-Model 111 (BIO-RAD) was used for isoelectric focusing (IEF) and defining isoelectric points (pI) of enzyme isoforms. IEF was carried out in 7.5% polyacrylamide gel with 5.0% ampholyte on a pH gradient from 3.0 to 10.0 using IEF marker (pI range from 3.6 to 9.3; Sigma-Aldrich) [6]. MnP and MnIP isoenzymes were visualized by gel incubation in 4-Cl-1-naphtol/H_2_O_2_/potassium phosphate buffer solution with or without MnSO_4_, respectively, at room temperature till appearance of dark-brown bands. The laccase bands were located by dyeing into ABTS/phosphate buffer solution. After focusing, the gel was fixed in 12.0% trichloroacetic acid and protein bands were detected by staining with 0.1% Coomassie Brilliant Blue R (CBB R) in fixative (methanol, acetic acid, and H_2_O in ratio 45:10:45).

### Determination of hemicellulose, cellulose and lignin contents

Determination of hemicellulose, cellulose and lignin contents in untreated and fungal-treated wheat straw was carried out using modified methods of Kirk and Obst [12] and Van Soest et al. [13]. The dried (at 50 °C) and finely ground samples were treated by neutral-detergent solution (NDS), boiled and refluxed in order to remove the soluble sugars, proteins, pectin, lipids and vitamins from them. Acid-detergent solution (ADS) and 72% H_2_SO_4_ were used for removal the hemicellulose and cellulose from the residues and determination of lignin content. The difference in weight of the samples treated with NDS and ADS represents the hemicellulose content. Lignin content (LC) was defined by Klason method by incubation of sample obtained after ADS treatment with 72% H_2_SO_4_ at 30 °C and hydrolysis at 120 °C, and expressed as percentage of that present in fungal-untreted sample. Cellulose amount presented difference in mass of the ADS-treated sample and LC. The assays were carried out in four replications and the results are expressed as a mean ± standard error.

## Results

**Fig. 1:**
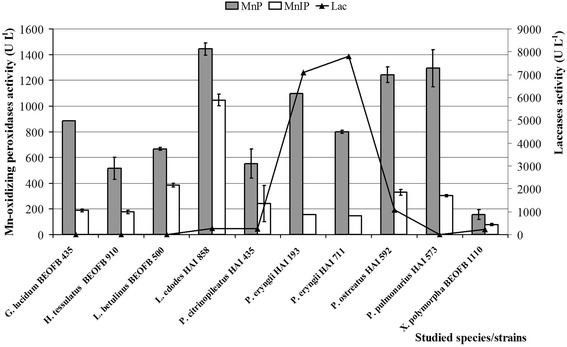
The activity of Mn-oxidizing peroxidases and laccases produced by selected fungal species after 21 days of solid-state fermentation of wheat straw

The selected fungal species have shown the ability to secrete Mn-oxidizing peroxidases and laccases during solid-state fermentation of wheat straw. The obtained results demonstrated a significant inter- and intraspecific diversity in enzymes production among studied fungal species/strains (Fig. 1).

The peak of MnP activity (1443.2 U L^−1^) was observed in *Lentinus edodes* HAI 858 and slightly lower values were noted in *Pleurotus pulmonarius* HAI 573 (1294.2 U L^−1^), *P. ostreatus* HAI 592 (1243.7 U L^−1^) and *P. eryngii* HAI 193 (1096.0 U L^−1^). The level of MnIP activity was slightly lower and the maximum was also noted in *L. edodes* HAI 858 (1045.5 U L^−1^). Generally, levels of laccase activity were remarkable higher than levels of Mn-oxidizing peroxidases activity, and the maximum value was obtained for *P. eryngii* HAI 711 (7804.3 U L^−1^) (Fig. 1).

The total protein content affected profiles of specific enzyme activities. The maximum MnP and MnIP specific activities were detected during wheat straw fermentation by *Trametes hirsuta* HAI 300 (110.4 U mg^−1^) and *L. edodes* HAI 858 (48.7 U mg^−1^), respectively, while the maximum specific laccase activity was noted in *P. ostreatus* HAI 592 (47.4 U mg^−1^).

**Fig. 2:**
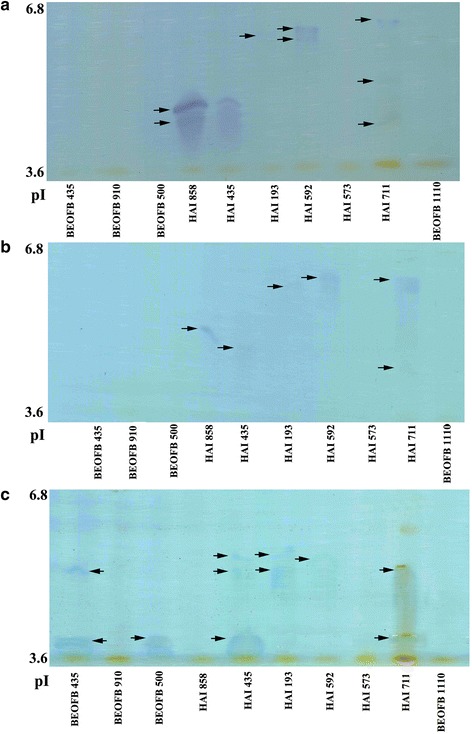
Isoelectric focusing pattern of Mn-dependent peroxidases (**a**), Mn-independent peroxidases (**b**) and laccases (**c**) obtained after 21 days of solid-state fermentation of wheat straw by selected fungal species

The IEF profiles of MnP, MnIP and laccase isoforms were variable depending on species/strain (Fig. 2). In the case of MnP, one very strong (pI about 4.6) and a few weak isoforms were visualized in *L. edodes* HAI 858 and *P. citrinopileatus* HAI 435, while in *P. ostreatus* HAI 592 two bends of pI 5.9 were observed. The intraspecific diversity was demonstrated in *P. eryngii*, i.e. in the starin HAI 193 only one weak bend of pI about 5.9 was noted while in the strain HAI 711 three isoforms of pI between 4.6 and 5.9 were separated (Fig. 2a). One MnIP bend of pI in the range between 3.6 and 5.9 was visualized in all tested species/strains, except *P. eryngii* HAI 711 where a few isoforms of pI about 5.9 appeared (Fig. 2b). Laccase zymogram has confirmed that the enzyme was the dominant ligninolytic enzyme in studied species, i.e. the most numerous strong isoforms were separated (Fig. 2c). Three laccase bends, one of pI 3.6 and two of pI about 4.6, were visualized in *P. citrinopileatus* HAI 435, two bends (pI of 3.6 and 4.6) were observed on *Ganoderma lucidum* BEOFB 435 and *P. eryngii* HAI 193 and HAI 711 zymograms, while other studied species and strains produced this enzyme in only one isoform.

### Lignocellulose degradation

The selected fungal species showed variable capacity of wheat straw delignification, as well as different levels of selectivity in polymers’ degradation (Table 2). *G. lucidum* BEOFB 435, *P. citrinopileatus* HAI 435 and *L. edodes* HAI 858 were the best delignifiers (58.5%, 56.0%, and 55.7%, respectively), while *Lenzites betulinus* BEOFB 500 was the weakest one which broke down only 33.5% of wheat straw lignin. *G. lucidum* BEOFB 435 was also the most effective hemicellulose degrader (74.8%) and the most selective degrader because it decayed only 0.7% of cellulose. Significant degradation selectivity was also obtained in wheat straw fermentation with *P. eryngii* HAI 711, *P. ostreatus* HAI 592, *P. pulmonarius* HAI 573 and *L. betulinus* BEOFB 500, while the lowest selectivity was noted for *Xylaria polymorpha* BEOFB 1110 which degraded lignin, hemicellulose and cellulose in the ratio of 42.9%:54.9%:44.0% (Table 2).

**Table 2:** The level of lignocellulose degradation by studied species/strains

Studied species	Strain code	Level of polymers degradation [%]		
		Lignin	Hemicellulose	Celullose
*Ganoderma lucidum*	BEOFB 435	58.5	74.8	0.7
*Hypsizygus. tessulatus*	BEOFB 910	49.4	30.6	20.0
*Lentinus edodes*	HAI 858	55.7	12.4	16.6
*Lenzites betulinus*	BEOFB 500	33.5	56.4	2.9
*Pleurotus eryngii*	HAI 193	36.7	2.1	1.4
	HAI 711	34.2	51.6	0.5
*Pleurotus pulmonarius*	HAI 573	50.4	15.3	3.8
*Pleurotus ostreatus*	HAI 592	40.3	35.8	0.8
*Pleurotus citrinopileatus*	HAI 435	56.0	30.7	29.2
*Xylaria polymorpha*	BEOFB 1110	42.9	54.9	44.0

## Discussion

Profiling ligninolytic potential of numerous insufficient studied fungal species, focused on the correlation between enzymes’ activity and polymers’ degradation extent, is the main contribution of this study. Previous results showed that important factors such as type of cultivation, composition of the plant material (especially the content of lignin in relation to hemicellulose and celullose), as well as cultivation conditions determine expression of ligninolytic enzymes [14–18]. However, the genetic basis of fungal species/strains has the essential role in ligninolytic enzyme synthesis. The existence of significant inter- and intraspecific variabilities in activities of studied ligninolytic enzymes as well as in the rate of lignin mineralization were in accordance with previous results. Thus, Camarero et al. [19] Stajić et al. [20] and Ćilerdžić et al. [21] showed interspecific diversity in MnP and laccase activity between *Coriolus hirsutus* and *C. pubescens*, *G. lucidum* and *G. carnosum*, and among numerous *Pleurotus* species, as well as in the delignification extent during solid-state fermentation of wheat straw with four *Pleurotus* species (*P. pulmonarius*, *P. floridanus*, *P. ostreatus* and *P. sajor-caju*). Silva et al. [11], Camarero et al. [19], Stajić et al. [20], Manavalan et al. [22] and Ćilerdžić et al. [23] also reported remarkable level of intraspecific diversity in laccase and MnP activities into *Cerrena maxima, G. lucidum*, and numerous *Pleurotus* species*.* Thus, Silva et al. [11] noted considerable diversity in activity of laccase produced during submerged wheat bran fermentation with four *G. lucidum* strains, i.e. activities were ranged from 0.58 U L^−1^ to 49,519 U L^−1^. After 14 days of wheat straw submerged fermentation with four *G. lucidum* strains studied by Ćilerdžić et al. [23] laccase activity was in the range from 153.5 U L^−1^ in strain BEOFB 432 to 5921.5 U L^−1^ in strain BEOFB 434. Likewise, activity of MnP produced during 21-old day solid-state fermentation of sugarcane bagasse by *G. lucidum* strain studied by Manavalan et al. [22] was significantly lower in comparison with value reported for *G. lucidum* BEOFB 435 (70 U g^−1^ substrate vs. ~900 U 2 g^−1^ of substrate). Intraspecific variability in ligninolytic enzymes activities during submerged fermentation of mandarin peels with *G. applanatum* and *Trametes versicolor*, as well as solid-state fermentation of grapevine sawdust with numerous strains of *Pleurotus* species and wheat straw with *L. edodes* and *T. versicolor* was also demonstrated by Stajić et al. [20] and Songulashvili et al. [24]. Numerous results also demonstrated significant differences in MnP and laccase activity among *P. ostreatus* strains originated from various areas and cultivated on various plant raw materials under submerged or solid-state conditions [1, 25, 26]. Thus, in submerged fermentation of sugarcane bagasse, Dong et al. [25] noted MnP activity of even 150,000 U L^−1^ and absence of laccase production, while in cultivation on wheat bran under the same conditions strain studied by Sergentani et al. [1] synthetized highly active laccase (4730 U L^−1^) but not MnP. On the other hand, solid-state cultivation induced well secretion of the enzymes independently on substrate. Namely, during potato peels fermentation Ergun and Urek [26] obtained remarkable activities of both MnP and laccase (about 1000 U L^−1^ and 2000 U L^−1^, respectively) which were similar to values obtained after wheat straw fermentation by strain HAI 592 (1243.7 and 1085.9 U L^−1^, respectively). However, in the case of *Xylaria polymorpha* diversity in laccase activity between strain studied by Liers et al. [27] and strain BEOFB 1110 was remarkable, i.e. value obtained after submerged tomato juice fermentation was almost 5-fold higher than after solid-state wheat straw fermentation.

Species/strain genetical basis as well as cultivation conditions also affected isoform profile of the ligninolytic enzymes. Contrary to only two laccase isoforms produced during cultivation of *G. lucidum* in malt medium [28] and three isoenzymes in Korean strain grown in liquid glucose/peptone/yeast extract medium [29], Manavalan et al. [22] visualized even five isoforms in strain cultivated on solid sugarcane bagasse substrate. However, Stajić et al. [10] and Ćilerdžić et al. [23] demonstrated higher effect of genetic on isoenzyme profile. Namely, two *G. lucidum* strains of different origin, HAI 447 and BEOFB 431, synthetized four and three isoforms, respectively, in cultivation under the same conditions, in solid-state wheat straw/NH_4_NO_3_ medium. In the case of MnP, strain studied by Manavalan et al. [22] produced one isoform of pI 4.19 on sugarcane bagasse, strain BEOFB 431 synthetized two isoforms of pI 3.6 and 3.7 after submerged fermentation of wheat straw [6], while any isoform no visualized after 21-day old solid-state fermentation of that raw material with strain BEOFB 435. Inter- and intraspecific diversity in isoenzyme profile was also demonstrated within genus *Pleurotus*. Namely, Muñoz et al. [30, 31] reported two laccase bands in *P. eryngii* cultivated in glucose/ammonium-tartrate medium, two isoforms were separated after submerged fermentation of mandarine peels with strain HAI 616 [32], while strains studied in that study (HAI 193 and HAI 711) produced three and two isoenyzmes, respectively, during growth on solid wheat straw based medium. In the case of *P. ostreatus*, contrary to four laccase isoforms (POXA1b, POXA1w, POXA2, and POXC) separated by Guardina et al. [33] and Palmieri et al. [34], three ones were sinthetized by strains HAI 493 and HAI 494 after cultivation on grapevine sawdust [32], while any isoform was visualized after solid-state wheat straw fermentation with strain HAI 592.

The numerous reports also showed considerable diversity among species and strains in delignification capacity and polymer degradation selectivity [6, 25, 35]. Species studied in this study were better delignifiers than numerous other previously tested. Thus, *G. lucidum* BEOFB 435 degraded in approximatelly 38% more lignin than strain BEOGB 432 and about 35% more than *G. applanatum*, while the difference was higher within *P. eryngii*, i.e. strains HAI 193 and HAI 711 were more than twice better delignifiers than strain HAI 507 [6, 35]. In the case of *P. ostreatus*, strain HAI 592 was more selective degrader of wheat straw under solid-state cultivation than strain studied by Dong et al. [25] which submergedly fermented sugarcane bagasse (40.3% of lignin, 35.8% of hemicellulose and 0.8% of cellulose vs. 50%:35%:5%).

## Conclusion

During wheat straw fermentation, *Lentinus edodes* HAI 858 produced the most active Mn-oxidizing peroxidases and *Pleurotus eryngii* HAI 711 the most active laccase, which was confirmed by obtained zymograms. Visualized enzymes’ isoforms also demonstrated that laccases were the dominant ligninolytic enzymes in studied species. *Ganoderma lucidum* BEOFB 435 and *Pleurotus pulmonarius* HAI 573 were the most selective lignocellulose degraders. The studied white-rot fungal species have high capacity to degrade selectively lignocellulose and owing to that ability could be important participants in the processes of bioconversion of plant biomas in food, feed, paper and biofuels.
